# The effect of sampling window size on topographical maps of foveal cone density

**DOI:** 10.3389/fopht.2024.1348950

**Published:** 2024-04-09

**Authors:** Emma Warr, Jenna Grieshop, Robert F. Cooper, Joseph Carroll

**Affiliations:** ^1^ Department of Ophthalmology & Visual Sciences, Medical College of Wisconsin, Milwaukee, WI, United States; ^2^ Joint Department of Biomedical Engineering, Marquette University and Medical College of Wisconsin, Milwaukee, WI, United States; ^3^ Department of Cell Biology, Neurobiology & Anatomy, Medical College of Wisconsin, Milwaukee, WI, United States

**Keywords:** fovea, cone density, cone spacing, adaptive optics, retina

## Abstract

**Purpose:**

To characterize the effect of sampling window size on maps of foveal cone density derived from adaptive optics scanning light ophthalmoscope (AOSLO) images of the cone mosaic.

**Methods:**

Forty-four AOSLO-derived montages of the foveal cone mosaic (300 x 300µm) were used for this study (from 44 individuals with normal vision). Cone photoreceptor coordinates were semi-automatically identified by one experienced grader. From these coordinates, cone density matrices across each foveal montage were derived using 10 different sampling window sizes containing 5, 10, 15, 20, 40, 60, 80, 100, 150, or 200 cones. For all 440 density matrices, we extracted the location and value of peak cone density (PCD), the cone density centroid (CDC) location, and cone density at the CDC.

**Results:**

Across all window sizes, PCD values were larger than those extracted at the CDC location, though the difference between these density values decreased as the sampling window size increased (p<0.0001). Overall, both PCD (r=-0.8099, p=0.0045) and density at the CDC (r=-0.7596, p=0.0108) decreased with increasing sampling window size. This reduction was more pronounced for PCD, with a 27.8% lower PCD value on average when using the 200-cone versus the 5-cone window (compared to only a 3.5% reduction for density at the CDC between these same window sizes). While the PCD and CDC locations did not occur at the same location within a given montage, there was no significant relationship between this PCD-CDC offset and sampling window size (p=0.8919). The CDC location was less variable across sampling windows, with an average per-participant 95% confidence ellipse area across the 10 window sizes of 47.56µm² (compared to 844.10µm² for the PCD location, p<0.0001).

**Conclusion:**

CDC metrics appear more stable across varying sampling window sizes than PCD metrics. Understanding how density values change according to the method used to sample the cone mosaic may facilitate comparing cone density data across different studies.

## 1 Introduction

Adaptive optics scanning light ophthalmoscopy (AOSLO) enables direct observation of the human photoreceptor mosaic with single-cell resolution (1–3). Of particular interest are the cone photoreceptors at the fovea – a highly specialized region of the retina that supports our high-acuity vision (4, 5). Relative to the parafoveal cone mosaic, foveal cone density is higher and the cones are contiguously packed (due in part to the absence of rod photoreceptors) (6). Given the vital importance of foveal cone photoreceptors for our vision, and the involvement of the fovea in many pathologies, there is significant interest in advancing quantitative biomarkers to assess the foveal cone mosaic. Numerous metrics exist for describing geometric properties of the cone mosaic, though cone density and cone spacing are used most often (7–10). Despite convergence on these metrics to characterize the foveal cone mosaic, there remain differences in the methods used to derive them – limiting the ability to compare results from different studies and ultimately limiting progress toward the development of robust clinical biomarkers.

A frequent inconsistency between studies relates to the size and shape of the sampling window used to derive metrics of the cone mosaic. The steep cone density gradient in the central fovea requires special consideration of how large of an area to use when deriving a local estimate of cone density (6). Larger sampling windows will result in lower values of peak density due to inclusion of lower density neighboring regions, while smaller sampling windows result in more noisy density maps that can make derivation of the global peak cone density (PCD) location challenging. Despite this, fixed-area sampling windows have been used in many studies (6, 8, 9, 11–20). This issue is partially overcome by using sampling windows that include a fixed number of cones, regardless of the retinal location. While this approach has been used by some investigators (10, 21–24), there is inconsistency in the number of cones sampled. Here we examined the relationship between the number of cones included in the sampling window and cone mosaic metrics (location and value of PCD, location of the cone density centroid (CDC), and density at the CDC). We also report the effect of variable sampling windows on the overall cone density topography for individual participants.

## 2 Methods

### 2.1 Participants

This study followed the tenets of the Declaration of Helsinki and was approved by the Institutional Review Board at the Medical College of Wisconsin (PRO 30741). Included in this study were right eyes of 44 participants (age range: 12-61 years; 17 males and 27 females) with self-reported normal vision and previously imaged as part of Cava et al. (2020) and Wynne et al. (2022) (23, 24). Prior to AOSLO imaging, axial length (IOL Master, Carl Zeiss Meditec, Dublin CA, USA) and autorefraction (KR-800S, Topcon Corporation, Tokyo, Japan) measurements were taken. The study eye was then dilated and accommodation suspended with one drop of 2.5% phenylephrine hydrochloride (Akorn, Lake Forest, IL, USA) and one drop of 1% tropicamide (Akorn, Lake Forest, IL, USA) for participants aged 18 years and older. For participants under age 18, one drop of 1% cyclopentolate hydrochloride (Cyclomydril, Alcon Laboratories Inc., Fort Worth, TX, USA) was used for dilation and accommodation suspension.

### 2.2 AOSLO imaging, processing, and montaging

A dental impression bite bar was used for head stabilization during AOSLO imaging. Per our imaging protocol, confocal AOSLO videos consisting of 150-200 frames each were acquired at various locations within the fovea. Videos were taken at 1.5° field of view (FOV) with approximately 1° overlap between video locations or at 1.0° FOV with approximately 0.5° overlap. A 775nm or 790nm super luminescent diode (SLD) was used for imaging. Several techniques were used to improve the resolution of foveal cones during imaging. These techniques included imaging with 0.75° and 0.5° FOVs, using a 680nm SLD (incident power: 32.5µW), or using a sub-airy disk pinhole (0.5-0.7 Airy disk diameter) (24).

Each video was processed to create high-resolution .tif images as previously described (24). From each video, a minimally distorted reference frame was automatically chosen (25) and used to register and average the remaining frames in the video with a strip-based registration algorithm (25, 26). Frames were then repaired to remove additional distortion with a de-warping repair script (https://github.com/OCVL/Eye-Motion-Repair) based on a previous method (27). This repair script works by estimating the bias of random eye motion distortions throughout the reference frames, based on the median translation observed in each row of the registered images. The median translation fixes the distortion of the frame in a same-magnitude but opposite-direction approach (24, 28). These processing techniques create a .tif image with high signal-to-noise ratio from each acquired video. Additional improvement in the resolution of the central most foveal cones was possible by averaging multiple processed images from videos acquired at the same retinal location – either at different focal planes or simply at different time points. This approach reduces the between cell variation in reflectivity, making discrimination of cones in the mosaic easier (24, 29).

The scale of each montage (µm/pixel) was determined from the AOSLO system scale as well as the individual’s axial length (2) at the time of image acquisition. This calculation was previously described (19) using the following equation:


$$AOSLO\;Scale=\frac{T}{f_{1}T_{s}}\left(\frac{180}{\pi }\right)RMF\left(\frac{AL}{24}\right)$$

where T represents the periodicity of a Ronchi ruling (µm/cycle), f_1_ represents the focal length of the model eye in our system (µm), T_s_ represents the sampling period of the lines in the model eye image of the Ronchi ruling (pixels/cycle), RMF represents the assumed retinal magnification factor (291 µm/degree) of an eye with a 24.0mm axial length (30), and AL is the participant’s axial length (mm).

A custom MATLAB script (https://github.com/BrainardLab/AOAutomontaging) was used to automatically align the processed .tif images (31) into a single montage. Alignment of overlapping images was manually corrected using Adobe Photoshop CS6 (Adobe Systems, Inc., San Jose, CA, USA). Individual frames were blended to create a flattened, seamless image of the area containing the subjective peak cone density as well as its adjacent frames. Finally, a 300 x 300µm region of interest (ROI) centered at the subjective location of approximate highest cone density (locus of smallest cones/tightest packing) was cropped for analysis.

### 2.3 Cone counting and density mapping

**Figure 1:**
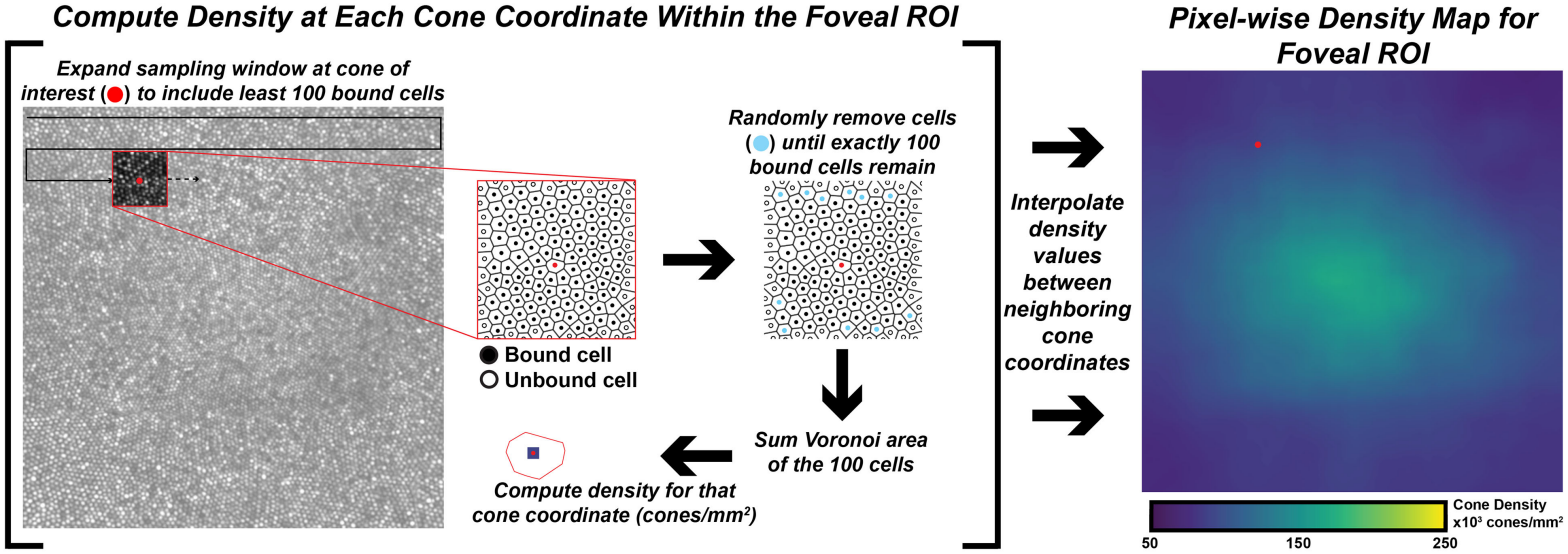
Extracting pixel-wise density from a foveal montage. Shown in the left-image is a 300 x 300µm foveal montage from participant JC_11660. Following cone identification, we calculate density at each cone coordinate in the image using a custom MATLAB script (https://github.com/AOIPLab/Metricks/releases/tag/Warr_et_al_2024). The script works by centering a square sampling window on a given coordinate and expanding the window until at least the specified number of bound Voronoi cells (e.g., 5, 10, 15, 20, 40, 60, 80, 100, 150, 200 cones) is included. An example of a 100-cone sampling window is shown in the red-outline box within the foveal montage. The center coordinate is identified with a red circle, unbound cells are identified with an open circle, and bound cells are identified with a black circle. In some instances, the window includes more than the specified number of cells. When this occurs, the script randomly removes excess coordinates (cyan circles) to achieve the exact number of specified bound cells. Then, density for the center coordinate is calculated by dividing the number of coordinates in that window by the sum of their Voronoi areas. The process is repeated until a density value is calculated at every coordinate of the montage, from which an interpolated density map is generated (right-most image).

Cones were identified using a semi-automated cone counting software (Mosaic Analytics; Translation Imaging Innovations, Hickory, NC, USA). One experienced human grader (JC) manually corrected the cone markings automatically identified by the algorithm. For each foveal montage, a cone coordinate file was generated containing the (*x,y*) locations of the individual cone markings. For this study we analyzed the effect of sampling window size when determining bound cone density for individual foveal montages. We used a square sampling window and specified the number of cells to be included in each window using a custom density mapping script (https://github.com/AOIPLab/Metricks/releases/tag/Warr_et_al_2024). The criterion sampling window size for generating the density maps was adjusted to include 5, 10, 15, 20, 40, 60, 80, 100, 150, or 200 cells. The sampling window expands at each coordinate until it includes at least the specified number of bound Voronoi cells. Due to the irregularity of Voronoi cells at the fovea, the sampling window is often unable to be sized such that it exactly matches the specified number of bound Voronoi cells. In these instances, we set the sampling window to the smallest size that still exceeds the specified number of cells, then randomly remove Voronoi cells around the edge of the sampling window until the exact number of specified cells is achieved. For edge cells in the montage, the sampling window expands on two or three sides toward the center of the montage instead of expanding proportionally from the target coordinate (this way, density is only calculated within the bounds of the montage). The density at each coordinate is calculated by taking the number of bound cells divided by the sum of their Voronoi areas. The window moves to each coordinate in the coordinate file and a pixelwise linear interpolated density map is created for each montage (**Figure 1**).

**Figure 2:**
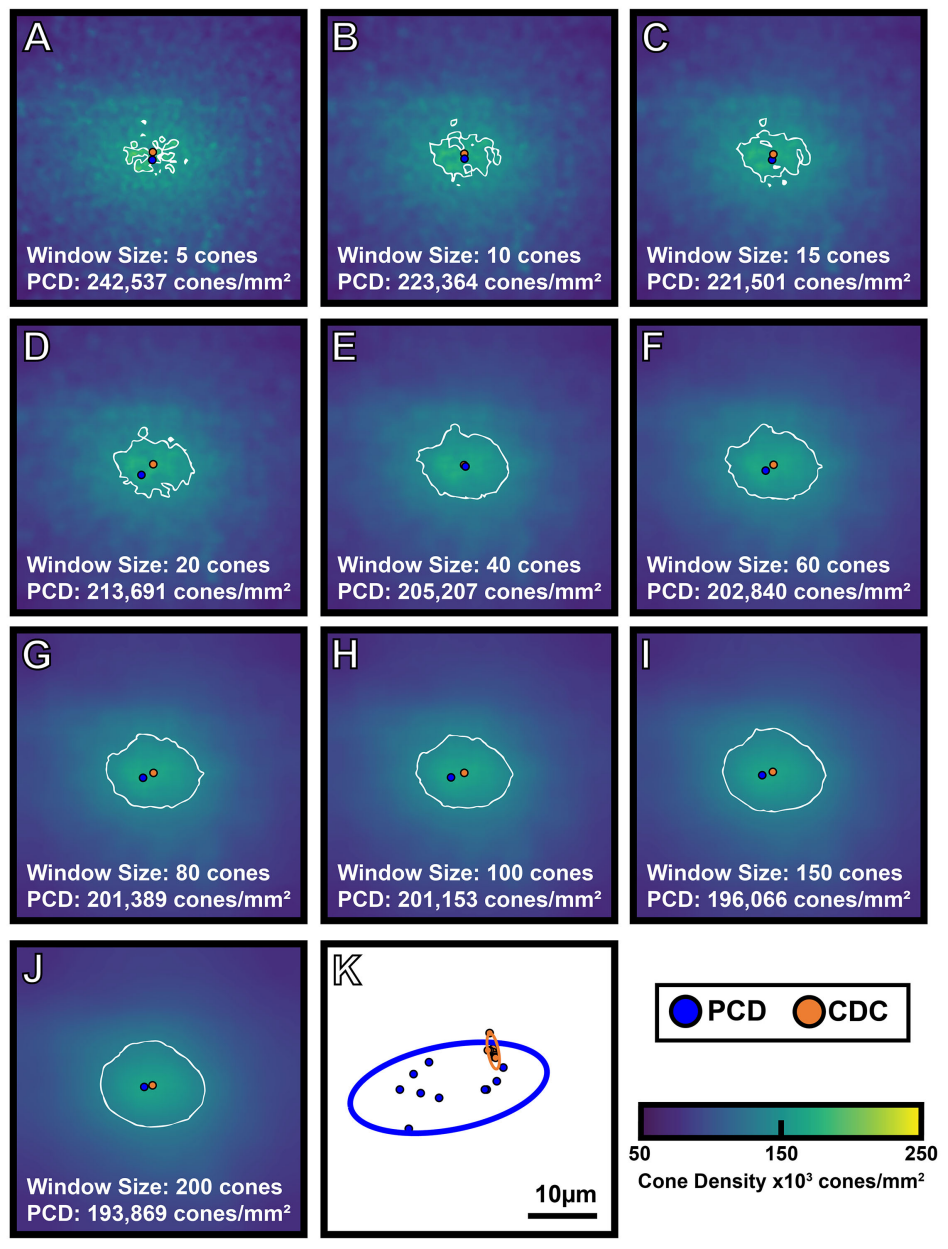
Visualizing the effect of sampling window size on foveal cone topography. A 300 x 300µm foveal montage was cropped from participant JC_11686 and cone coordinates were semi-automatically identified (*see Methods*). Density maps were generated using 10 different sampling window sizes, with corresponding cone density maps shown in **(A-J)**. As window size increases, PCD decreases. Also, as window size increases, the density maps show a smoother gradient of density change from the foveal center to the perifovea. The blue filled circle represents the location of PCD, while the orange filled circle represents the location of the CDC in each density map. The white outline represents the 80% isodensity contour. Shown in panel **(K)** are the individual locations of PCD and CDC produced by each of the 10 sampling windows for this participant (blue and orange filled circles, respectively). The blue outline represents the 95% confidence ellipse of the PCD locations, while the orange outline represents the 95% confidence ellipse of the CDC locations. The area of the 95% PCD confidence ellipse is 274.65µm² while the area of the 95% CDC confidence ellipse is 6.34µm². The CDC locations were less affected by the sampling window size, reflected by the smaller 95% confidence ellipse.

The interpolated pixel in each foveal montage containing the absolute maximum density is the location of the PCD and the subsequent density value at that point is taken as the PCD. The CDC was determined by generating an 80% isodensity contour of the region that includes pixels containing the top 20^th^ percentile of density values within the foveal montage. We then fit an ellipse around this 80% isodensity region and define the center of this best-fit ellipse as the CDC location (see **Supplementary Figure 1**). This method for determining the CDC is based off the method described by Reiniger et al. (2021) (32). We determined PCD and CDC metrics from each sampling window condition across all 440 density maps (44 participants with 10 density maps each). The 10 density maps for one participant are shown in **Figure 2**. To assess intra-individual reproducibility of the PCD and CDC locations, we computed the 95% confidence ellipse areas for both PCD and CDC locations from all 10 window sizes using a custom MATLAB script (https://github.com/AOIPLab/Metricks/releases/tag/Warr_et_al_2024). Example PCD and CDC 95% confidence ellipses for a single participant are shown in **Figure 2K**.

### 2.4 Assessing overall cone mosaic topography

**Figure 3:**
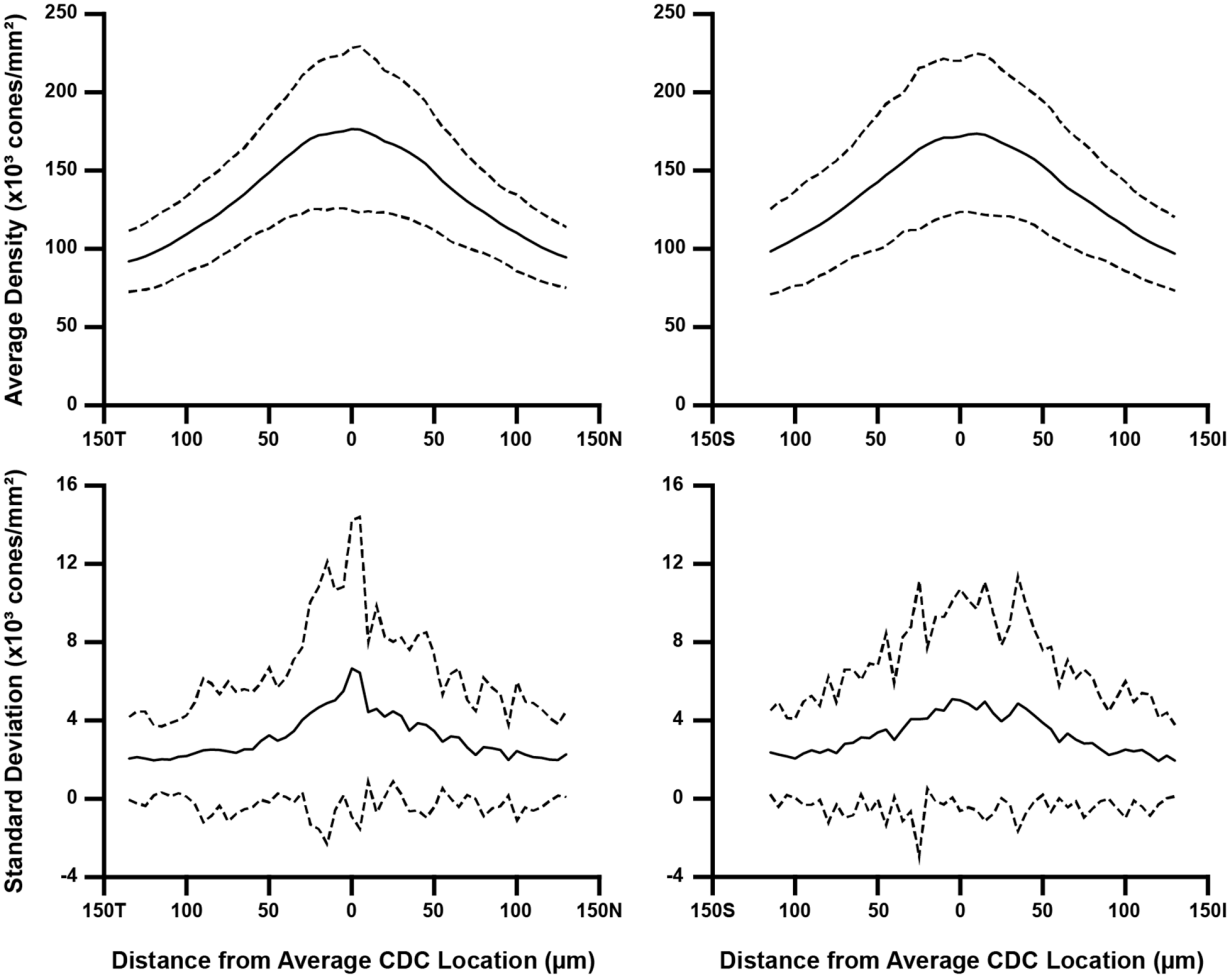
Topographical variation in foveal cone density. For each participant, we derived two composite maps (mean density and density standard deviation) using their individual density maps from the 10 sampling windows. Horizontal and vertical cross-sections of mean density and density standard deviation were then extracted at their average CDC point (0 on the x-axis). We averaged these data across all 44 participants, which is represented by the solid line in all four plots. Also plotted are ±2 standard deviations of all participant data (dashed lines). Data are shown separately for the horizontal (left) and vertical cross-sections (right). As seen in the top plots, the between-participant variance in average density is greatest near the average CDC point where the pixel-wise density is highest and changes most rapidly. Meridian orientation is indicated on the x-axes (T=temporal, N=nasal, S=superior, I=inferior).

To assess how the sampling window affects overall aspects of foveal cone topography (not just PCD and CDC), we created two separate composite maps (mean density and density standard deviation) for each participant using the data from their 10 individual density maps. From these two maps, we extracted a horizontal and vertical cross section through the average CDC location (derived from that participant’s 10 individual density maps). For most participants, the average CDC (*x,y*) coordinates were not whole numbers. In these cases, we used a weighted average of the values in the rows and/or columns neighboring the average CDC point (for example, if the average CDC *x*-coordinate was 150.3, the 150^th^ column of values were weighted at 70% while the 151^st^ column of values were weighted at 30%). Shown in **Figure 3** are topographical plots from both cross-sections illustrating the average trend from all 44 participants. All MATLAB scripts utilized above can be found at: https://github.com/AOIPLab/Metricks/releases/tag/Warr_et_al_2024.

### 2.5 Statistics

Linear units were used to represent PCD and CDC density (cones/mm²) as well as PCD and CDC locations (µm). We also repeated the comparisons using angular units to represent density (cones/deg²). See **Supplementary Table 1** for participant level data. A Shapiro-Wilk normality test was used to determine the use of parametric or nonparametric approaches. All statistics were calculated using GraphPad Prism (Prism 9.0.0; GraphPad Software, San Diego, CA, USA).

## 3 Results

**Figure 4:**
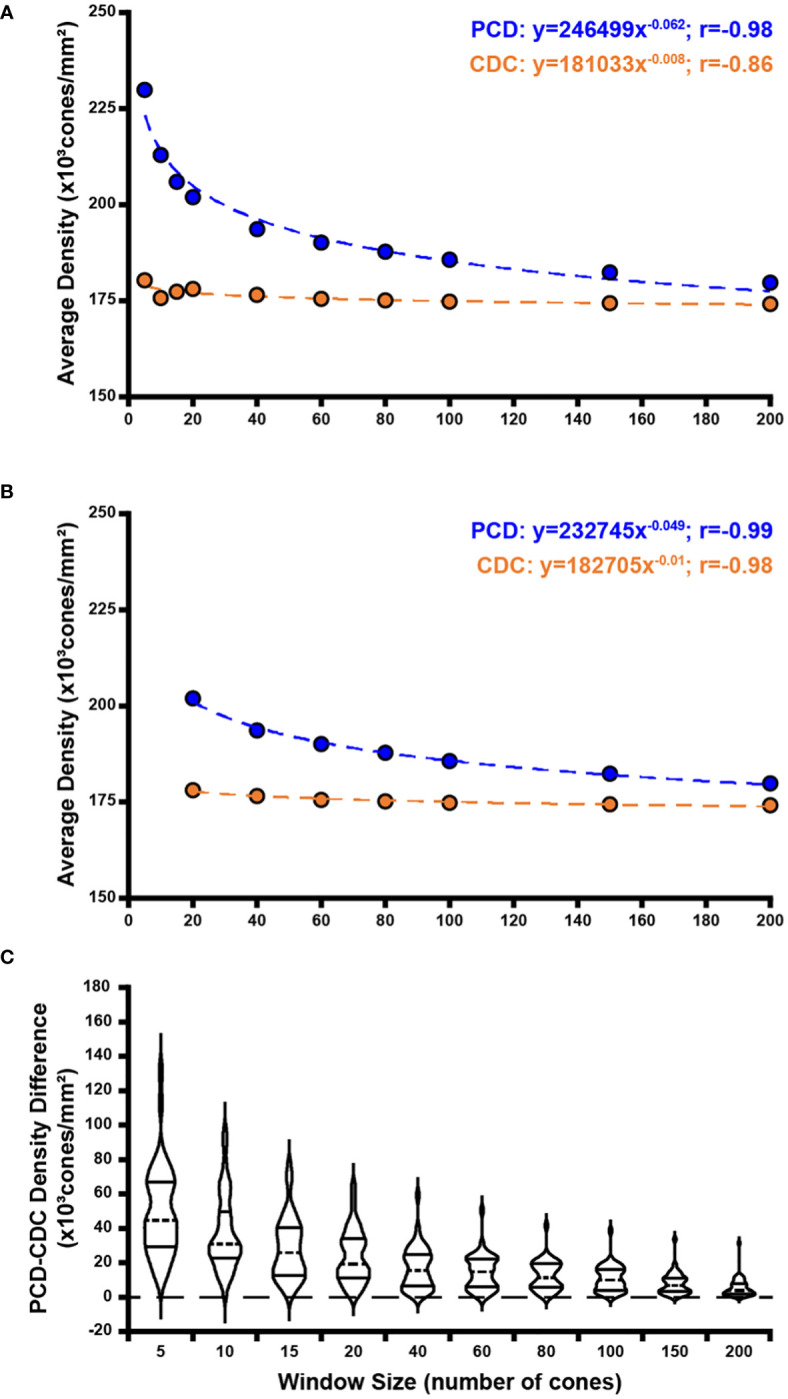
Effect of sampling window size on peak cone density (PCD) and cone density centroid (CDC) values. Shown in panel **(A)** are average PCD and average density at the CDC values at all 10 sampling windows across all 44 participants (blue and orange filled circles, respectively). The PCD and CDC values are each fit to a power function (best fit lines illustrated by dashed blue and orange lines, respectively). As sampling window size increases, PCD decreases precipitously while density at the CDC remains more stable (though CDC density values still showed a significant correlation with sampling window, r=-0.86). **(B)** We re-fit these data excluding the three smallest sampling windows (5, 10, 15 cones). The relationship between sampling window size and density value is stronger with the exclusion of smaller sampling windows which are more susceptible to oversampling density. Symbols and lines are same as in **(A)**. In all functions described, *x* is the sampling window size in number of cones and *y* is the cone density in cones/mm². **(C)** We computed the difference in PCD and CDC density for each participant at each window size. Each violin in the plot illustrates the distribution of density differences across participants (dashed line = median, solid lines = upper and lower quartile in each violin). Density difference decreases significantly as window size increases (p<0.0001, Kruskal-Wallis test).

Across all window sizes, average PCD values were larger than those extracted at the CDC location (p=0.0012, paired t test; **Figure 4A**). On average, PCD and CDC both decreased with increasing sampling window size, though the effect was larger for PCD (r=-0.8099, p=0.0045) than CDC (r=-0.7596, p=0.0108). As the density values at the CDC with the smallest window sizes behaved in an inconsistent fashion, we also examined the relationship between density and window size using only window sizes of 20 cones and larger (**Figure 4B**). We again see a strong relationship between PCD and window size (r=-0.9192, p=0.0034) and also for CDC density values (r=-0.8595, p=0.0132). As summarized in **Supplementary Table 2**, our density values are comparable to previous estimates in individuals with normal vision (10, 13, 21, 23, 24, 33–36). For example, with a window size of 100 cones, the average PCD from all 44 participants was 185,501 cones/mm² (range=128,007-244,921 cones/mm²) and the average density at the CDC was 174,696 cones/mm² (range=121,169-242,614 cones/mm²). At a window size of 150 cones, the average PCD from all 44 participants was 182,184 cones/mm² (range=126,323-239,362 cones/mm²) and the average density at the CDC was 174,295 cones/mm² (range=121,891-235,663 cones/mm²). The intra-participant differences in density estimates at the PCD and CDC locations decreased significantly as window size increased (p<0.0001, ANOVA, **Figure 4C**). This was driven largely by the increased PCD values obtained with smaller sampling windows (Tukey’s multiple comparisons test, see **Supplementary Table 3**). The same trends were observed when reporting density in angular units (cones/deg^2^), see **Supplementary Figure 2**. Across, the 44 participants, as the number of cones in the sampling window increase, we observed a proportional increase in window area (y=9.21x + 1.7146, R² = 1; where *x* is the window size in number of cones and *y* is the average bound area window size across all participants). For example, the average area for the 200-cone sampling window was 1,841.14µm² while the average area for a 5-cone sampling window was 45.98µm² (see **Supplementary Table 4**).

**Figure 5:**
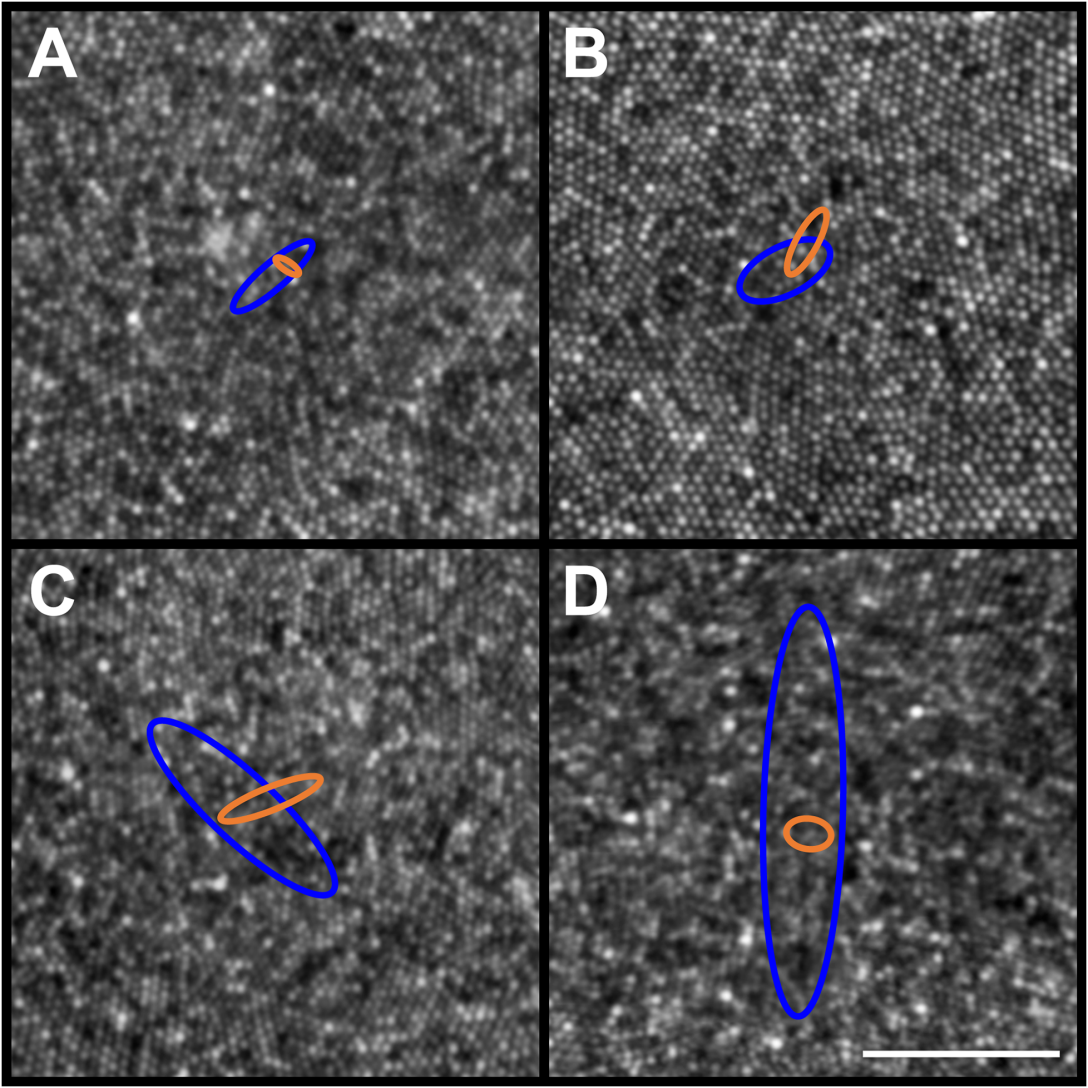
Example PCD and CDC 95% confidence ellipses from four participants (JC_11354, JC_11321, JC_11613, and JC_11631). Individual PCD and CDC 95% confidence ellipses are shown (blue and orange, respectively). Shown in panel **(A)** is JC_11354 who has an average PCD of 184,048 cones/mm²; average PCD ellipse area of 132.91µm^2^; and average CDC ellipse area of 20.13µm^2^. Shown in panel **(B)** is JC_11321 who has an average PCD of 153,724 cones/mm²; average PCD ellipse area of 226.70µm^2^; and average CDC ellipse area of 69.65µm^2^. Shown in panel **(C)** is JC_11613 who has an average PCD of 224,457 cones/mm²; average PCD ellipse area of 788.26µm^2^; and average CDC ellipse area of 110.78µm^2^. Shown in panel **(D)** is JC_11631 who has an average PCD of 200,331 cones/mm²; average PCD ellipse area of 1625.52µm^2^; and average CDC ellipse area of 60.70µm^2^. The center of each panel aligns with the center of the 300 x 300µm AOSLO montage, though note the 50µm scale bar (white line in panel **D**) indicating that these are zoomed in to reveal the small differences between ellipses. The area of the PCD 95% confidence ellipse is larger than the area of the CDC 95% confidence ellipse, with one exception (JC_11830; *see text*).

**Figure 6:**
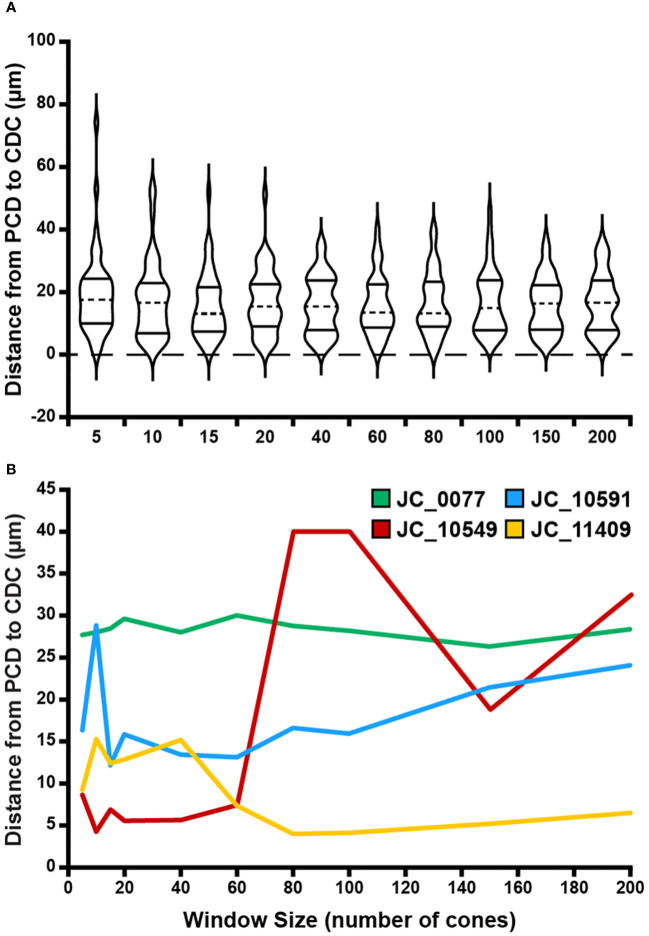
Effect of sampling window size on PCD and CDC location offset. **(A)** At a given window size, we determined the Euclidian distance between the location of the PCD and the location of the CDC for each participant. Each violin in the plot illustrates the distribution of PCD-CDC location offsets across all 44 participants (dashed line = median, solid lines = upper and lower quartile in each violin). Though the PCD and CDC locations occurred at different points within a given montage, no significant relationship was found between average PCD-CDC location offset and sampling window size (p=0.89). **(B)** Shown are examples highlighting the variable inter-individual variability in the relationship between PCD-CDC location offset and sampling window size. Some remain relatively constant (JC_0077), some showed convergence of the PCD and CDC location with increasing sampling window (JC_11409), some showed divergence (JC_10591), while others showed inconsistent trends (JC_10549).

In addition to density values, we observed variation in PCD and CDC location as a function of window size. Across all 440 ROIs (44 participants with 10 window sizes each), there were no instances where the reported PCD value occurred at more than one point. The average PCD confidence ellipse area across all participants was 844.10µm^2^, which was significantly greater than the average CDC confidence ellipse area (47.56µm^2^, p<0.0001, Wilcoxon matched-pairs signed-rank test). PCD 95% confidence ellipse areas were greater than CDC 95% confidence ellipse areas for all participants with one exception (JC_11830). Across the 44 participants, PCD 95% confidence ellipse area ranged from 105.28 to 8,389.90µm^2^, while CDC 95% confidence ellipse area ranged from 2.50 to 481.73µm^2^. Example 95% confidence ellipses are shown for four participants in **Figure 5**. There was variation between participants in the relationship between the magnitude of the offset between PCD and CDC location and sampling window size. On average, the 44 participants demonstrate relative stability in PCD-CDC location offset as sampling window size increases (**Figure 6A**). However, when assessed individually, some participants showed convergence of the PCD and CDC location with increasing sampling window, some showed divergence, while others showed inconsistent trends (**Figure 6B**). This heterogeneity in how window size impacts PCD-CDC offset is likely due to the underlying global topography of each cone mosaic.

Consistent with prior studies of cone topography (6, 22, 35), we observed a decline in average cone density as a function of eccentricity in both the horizontal and vertical meridians (**Figure 3**). Additionally, we observed greater within-participant standard deviation near the CDC location compared to locations toward the edge of the foveal ROI – indicating that the variability induced by using different sampling windows affects density more in areas of rapidly changing density (foveal center) compared to areas where density is more uniform (edge of the foveal ROI used here).

## 4 Discussion

Consistent with previous studies of the parafoveal cone mosaic (16, 37–39), our results highlight the importance of defining the sampling window used in deriving maps of foveal cone density. Importantly, we find that the value and location of PCD (versus CDC) is more significantly affected by using a variable sampling window size. This is demonstrated by the steeper decline in PCD as window size increases as compared to density at the CDC (**Figure 4A**). Additionally, the smaller average CDC 95% confidence ellipse area compared to PCD 95% confidence ellipse area demonstrates greater stability in CDC location compared to PCD location as sampling window size changes. These findings are expected based on how we define PCD versus density at the CDC. PCD is the interpolated point of absolute highest density in a given montage whereas density at the CDC describes a relative maximum based on a pooled region of highly dense locations. Thus, when the sampling window is small, we can resolve several high-density regions in the foveal mosaic, visible as small “islands” of 80% isodensity contour (**Figures 2A–D**). To derive the CDC, we generate a best fit ellipse of the 80% isodensity contour and define the center of that ellipse as the CDC location. This method identifies a point whose density is an average of a region of high cone density. Therefore, despite changes in sampling window size, the CDC location of an individual ROI remains more stable than the PCD location. As well, previous studies have demonstrated superior intersession repeatability for CDC location compared to PCD location (32) as well as superior intergrader reproducibility in CDC location relative to PCD location (23). Taken together, the emerging picture is that CDC represents a more robust metric to describe the “center” of the foveal cone mosaic.

As reported by Wang et al. (2019), use of smaller sampling windows may produce unreliable estimates of PCD and PCD location (34), though it is important to note they used a fixed dimension sampling window, which may contain variable numbers of cones across participants. Additionally, their sampling window of 10 arcmin contains over 200 cones, which would be larger than the largest sampling window used in our study. Our data support the notion that smaller window sizes impact PCD and we show that this also applies to the CDC (albeit to a lesser degree). When excluding the smallest sampling window sizes, we observe a strong relationship between sampling window size and both PCD and density at CDC (**Figure 4B**). This observation may allow comparison of density estimates between studies that used different sampling windows to construct their respective cone density maps. However, while this approach may work on average, there is variation between individual cone mosaics in how density estimates and locations vary as a function of sampling window size (**Figure 6B**). This is likely due to more global features of cone topography, including individual variation in kurtosis of the cone density versus eccentricity function. Further work is needed to understand if and how best to combine data across studies employing different methodology, as large and robust normative databases are needed to advance the use of AOSLO imaging.

Limitations of our study include reliance on a single observer’s cone markings – errors or biases in their coordinates may exist (23). As such, generalization of these trends should be applied carefully to other datasets where coordinates were derived from different observers. Additional factors related to sampling the cone mosaic could be considered in future studies – such as inclusion of “edge” cells within the window, shape of the window (square, arcuate, circular), and interpolation methods. Additionally, our sample of 44 participants was almost 2:1 female (27F, 17M) and almost exclusively white (approximately 70% of participants self-reported race as white). As it is unknown whether cone mosaic topography varies with race and ethnicity, our analysis should be repeated in a more heterogenous population.

Ultimately, the “cost” of variation in any metric (including those assessed here: PCD/CDC value and PCD/CDC location) depends on the subsequent application. For this reason, we do not identify one sampling window as the ideal size when extracting density metrics. Correlation of cone density with measures of function (e.g., acuity or sensitivity) may wish to utilize sampling windows with sizes comparable to some feature of the visual system (such as fixational stability) (34). Moreover, correlation with measures of function would be best made using cone density at the retinal location used for the specific task (40–42), which may not occur at the PCD or the CDC (32, 43, 44). Thus, in these scenarios, the PCD or CDC location may not be overly relevant. Likewise, correlation of mosaic metrics to other structural features in other imaging modalities would require co-registration of images to ensure alignment of the cone mosaic measures with these other retinal features (45, 46). One application that depends on having a reliable “anchor” within the cone mosaic is selecting parafoveal and perifoveal regions of interest from a larger montage for subsequent analysis. Whether PCD or CDC, this anchor serves as the (*0,0*) reference point from which retinal eccentricity of a given region of interest is computed (47). Though we observe an offset in PCD and CDC location at all window sizes, if choosing PCD or CDC as an anchor point this offset would remain a fixed value at all retinal eccentricities. However, as cone (and rod) density changes with eccentricity (6, 35), this fixed offset in the location assigned to a given region of interest could result in variably misleading conclusions about the health of the underlying mosaic. Our findings support the use of CDC as a more reliable anchor for such applications, even where resolution of the most densely packed cones is possible and PCD measures are desired for different purposes.

## Data availability statement

The raw data (foveal montages, cone coordinate files, density matrices) supporting the conclusions of this article will be made available upon request to the corresponding author (jcarroll@mcw.edu), without undue reservation.

## Ethics statement

The studies involving human participants were reviewed and approved by the Medical College of Wisconsin IRB. The studies were conducted in accordance with local legislation and institutional requirements. The participants (or when appropriate, their parent’s or guardian’s) provided their written informed consent to participate in this study. 

## Author contributions

EW: Conceptualization, Formal analysis, Visualization, Writing – original draft, Writing – review & editing. JG: Software, Writing – review & editing. RFC: Funding acquisition, Software, Writing – review & editing. JC: Conceptualization, Formal analysis, Funding acquisition, Supervision, Visualization, Writing – review & editing.
