# Supplementary material for: The effect of sampling window size on topographical maps of foveal cone density
Source: Front Ophthalmol (Lausanne). 2024 Apr 9;4:1348950. doi: 10.3389/fopht.2024.1348950 (PMC11182112; doi:10.3389/fopht.2024.1348950)
Supplement: Supplementary file 1 [file DataSheet_1.pdf]

Supplementary Material

The effect of sampling window size on topographical maps of foveal cone density

Emma Warr, Jenna Grieshop, Robert Cooper, Joseph Carroll*

*** Correspondence:** Joseph Carroll: jcarroll@mcw.edu

1 Supplementary Figures and Tables

1.1 Supplementary Figures

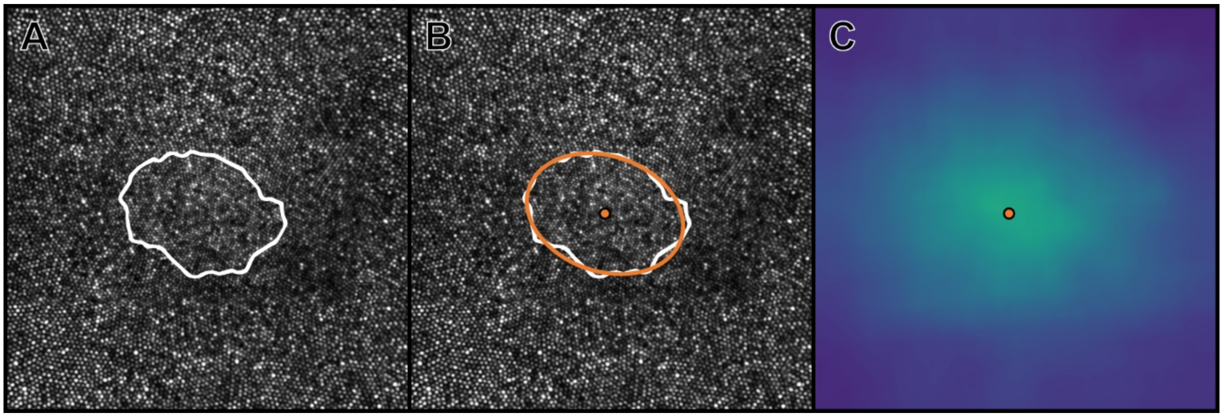

Supplementary Figure 1. Derivation of the cone density centroid (CDC). Shown in panel (**A**) is a 300 x 300µm foveal montage from participant JC_11660. Cone coordinates were semi-automatically identified and pixel-wise cone density was calculated using a custom Matlab script (*see methods* and **Figure 1**) https://github.com/AOIPLab/Metricks/tree/Warr_Revisions_Dev. The white outline represents the 80% isodensity contour encompassing the area of the montage containing the highest 20% of cone density. From this 80% isodensity contour, a best-fit ellipse is generated (orange ellipse shown in panel **B**). The center of this ellipse is the CDC location represented by an orange filled circle in panels (**B**) and (**C**). The cone density at this CDC location is density at the CDC. Finally, shown in panel (**C**), is a linear-interpolated density heat map of the foveal montage. The 80% isodensity contour, best-fit ellipse, and density map were all generated with a 100-cone sampling window. This process was repeated for all sampling windows for all subjects.

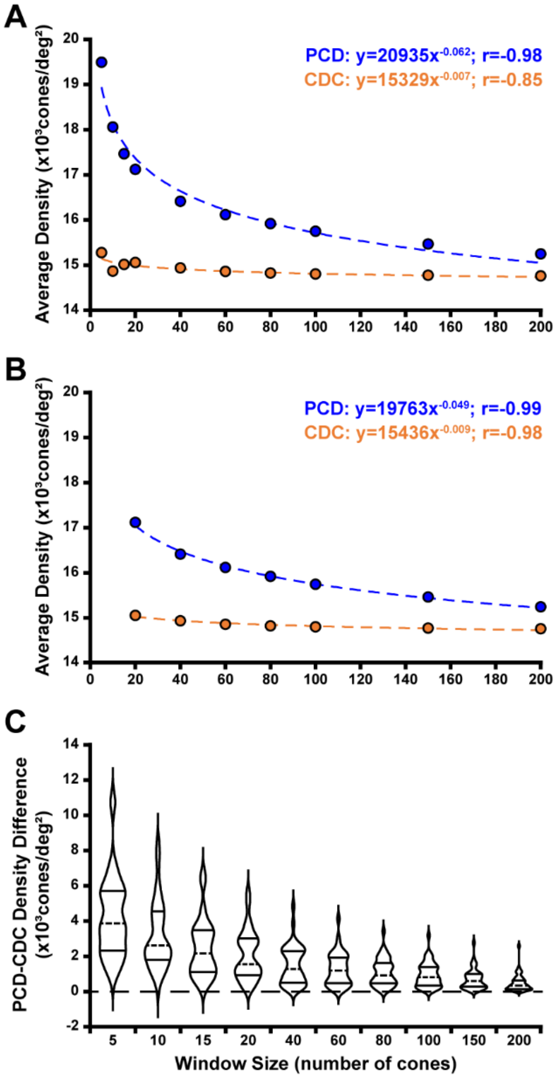

Supplementary Figure 2. Effect of sampling window size on peak cone density (PCD) and density at the cone density centroid (CDC) with angular density metrics (cones/deg²). Shown in panel (**A**) are average PCD and average density at the CDC values at all 10 sampling windows across all 44 participants (blue and orange filled circles, respectively). The PCD and CDC values are fit to a power function (best fit lines illustrated by dashed blue and orange lines, respectively). (**B**) We re-fit these data excluding the three smallest sampling windows (5, 10, 15 cones). The relationship between sampling window size and density value is stronger with the exclusion of smaller sampling windows which are more susceptible to oversampling density. Symbols and lines are same as in (**A**). In all functions described, x is the sampling window size in number of cones and y is the density metric (PCD or density at the CDC) in cones/deg². (**C**) We computed the difference in PCD and CDC density for each participant at each window size. Each violin in the plot illustrates the distribution of density differences across participants (dashed line = median, solid lines = upper and lower quartile in each violin). For panels (**A**), (**B**), and (**C**), these data follow nearly identical trends as seen with PCD and density at the CDC calculated in linear units (cones/mm²) shown in **Figure 4**.

Supplementary Table 1. Results for Each Participant								
Participant ID	Sex	Age (yrs)	Axial Length (mm)	Avg. PCD (cones/mm²)	Avg. PCD (cones/deg²)	Avg. CDC (cones/mm²)	Avg. CDC (cones/deg²)	Avg. 80% Isodensity Area (µm²)
JC_11655	M	18	25.28	199,168	18,713	174,338	16,380	8,068
JC_11685	F	25	25.21	209,329	19,559	181,192	16,930	9,358
JC_11441	F	25	23.2	167,749	13,274	152,010	12,029	10,116
JC_11295	F	30	22.91	215,905	16,660	206,211	15,912	3,760
JC_0077	F	14	24.15	180,240	15,454	165,927	14,227	5,797
JC_10312	M	18	27.06	148,026	15,935	119,670	12,883	8,618
JC_11867	M	25	24.11	214,019	18,290	190,593	16,288	6,006
JC_11610	M	24	23.95	200,675	16,923	168,337	14,196	6,012
JC_11467	F	61	23.63	227,349	18,663	165,057	13,550	7,871
JC_11442	M	25	23.71	258,332	21,350	225,308	18,621	3,787
JC_11068	F	32	22.36	190,248	13,984	155,499	11,430	10,739
JC_0616	M	30	24.24	214,754	18,551	199,737	17,254	5,910
JC_11857	F	23	23.29	165,228	13,176	143,397	11,435	8,604
JC_11660	F	24	23.07	210,331	16,457	199,016	15,572	6,152
JC_10145	F	53	24.72	117,946	15,986	155,625	13,981	10,852
JC_0007	M	42	27.5	167,011	18,568	144,348	16,049	12,183
JC_11613	F	28	24.94	224,457	20,525	199,981	18,287	6,627
JC_11350	M	34	23.1	205,858	16,149	186,924	14,664	9,243
JC_11159	F	29	23.65	179,787	14,784	172,968	14,223	5,617
JC_0878	F	12	24.03	188,316	15,987	175,149	14,869	7,708
JC_11591	M	22	24.44	194,537	17,083	187,552	16,470	5,186
JC_10567	F	27	22.32	178,663	13,085	168,557	12,345	5,340
JC_11354	F	24	24.53	184,048	16,281	178,750	15,813	7,649
JC_11321	F	30	23.75	153,723	12,748	144,857	12,012	7,376
JC_11686	F	23	23.51	210,361	17,094	194,992	15,845	4,312
JC_11631	M	25	25.63	200,331	19,347	157,344	15,195	8,642
JC_11666	M	25	24.76	220,790	19,900	194,697	17,548	8,239
JC_11444	F	27	24.03	202,436	17,185	171,862	14,590	9,599
JC_11584	M	27	24.69	198,934	17,828	166,392	14,912	9,519
JC_0200	M	30	24.47	175,251	15,427	145,597	12,817	8,567
JC_11409	F	23	23.48	215,067	17,431	204,114	16,544	5,804
JC_11314	F	23	24.05	132,765	11,290	128,489	10,926	8,189
JC_10339	F	30	23.55	176,845	14,419	167,669	13,671	8,089
JC_11923	F	24	23.48	183,179	14,847	169,601	13,746	7,061
JC_11617	F	48	24.06	191,333	16,283	180,364	15,350	5,404
JC_10591	M	28	23.58	239,241	19,556	208,116	17,012	7,770
JC_11575	F	28	22.93	212,631	16,436	187,223	14,472	8,095
JC_11830	F	49	25.48	203,848	19,457	162,028	15,465	5,519
JC_10220	F	28	22.93	186,297	14,400	172,811	13,358	4,479
JC_11469	M	27	23.23	266,354	21,131	248,199	19,691	4,164
JC_11658	F	24	22.18	227,433	16,449	212,667	15,381	3,657
JC_10549	M	26	23.98	192,729	16,293	174,983	14,793	9,717
JC_11372	M	32	24.32	156,654	13,622	140,116	12,184	18,427
JC_11103	F	55	24.46	209,324	18,412	198,647	17,473	7,929

Supplementary Table 2: Summary of Previous Studies on Foveal Cone Density

Reference	Cone Density Range	Sampling Window Size
Putnam *et al.* (2005)	114,963 – 226,929 cones/mm²	Circle; radius of 20.6µm
Li *et al.* (2010)	116,217 – 167,984 cones/mm²	Square; 150 cones
Zhang *et al.* (2015)	136,132 – 247,061 cones/mm²	Square; 5 x 5µm
Cooper *et al.* (2016)	ND<br>mean ± SD = 119,000 ± 23,300 cones/mm²	Square; 37 x 37µm
Wells-Gray *et al.* (2016)	ND<br>mean ±SD = 164,000 ± 24,000 cones/mm²	Square; 35 x 35µm
Wilk *et al.* (2017)	106,700 – 214,000 cones/mm²	Square; 37 x 37µm
Wang *et al.* (2019)	118,491 – 204,020 cones/mm²	Circle; 10 arcminutes
	123,611 – 208,606 cones/mm²	Circle; 7.5 arcminutes
Cava *et al.* (2020)	122,710 – 247,710 cones/mm²	Square; 100 cones
Domdei *et al.* (2021)	ND<br>13,733 – 18,406 cones/deg²	150 nearest neighbor cones
Reininger *et al.* (2021)	145,870 – 221,889 cones/mm²	150 nearest neighbor cones
Wynne *et al.* (2022)	117,626 – 220,011 cones/mm²	Square; 150 cones
Heitkotter *et al.* (2023)	125,520 – 249,297 cones/mm²	Square; 150 cones
Domdei *et al.* (2023)	147,038 – 215,681 cones/mm²	150 nearest neighbor cones

1. Putnam NM, Hofer HJ, Doble N, Chen L, Carroll J, Williams DR. The Locus of Fixation and the Foveal Cone Mosaic. J Vis (2005) 5(7):632-9.
2. Li KY, Tiruveedhula P, Roorda A. Intersubject Variability of Foveal Cone Photoreceptor Density in Relation to Eye Length. Invest Ophthalmol Vis Sci (2010) 51(12):6858-67.
3. Zhang T, Godara P, Blanco ER, Griffin RL, Wang X, Curcio CA, Zhang Y. Variability in Human Cone Topography Assessed by Adaptive Optics Scanning Laser Ophthalmoscopy. Am J Ophthalmol (2015) 160(2):290-300.
4. Cooper RF, Wilk MA, Tarima S, Carroll J. Evaluating Descriptive Metrics of the Human Cone Mosaic. Invest Ophthalmol Vis Sci (2016) 57(7):2992-3001.
5. Wells-Gray EM, Choi SS, Bries A, Doble N. Variation in Rod and Cone Density from the Fovea to the Mid-Periphery in Healthy Human Retinas Using Adaptive Optics Scanning Laser Ophthalmoscopy. Eye (2016) 30(8):1135-43. doi: 10.1038/eye.2016.107.
6. Wilk MA, Wilk BM, Langlo CS, Cooper RF, Carroll J. Evaluating Outer Segment Length as a Surrogate Measure of Peak Foveal Cone Density. Vision Res (2017) 130:57-66. doi: 10.1016/j.visres.2016.10.012.
7. Wang Y, Bensaid N, Tiruveedhula P, Ma J, Ravikumar S, Roorda A. Human Foveal Cone Photoreceptor Topography and Its Dependence on Eye Length. eLife (2019) 8:e47148. Epub 2019/07/28. doi: 10.1101/589135.
8. Cava JA, Allphin MT, Mastey RR, Gaffney M, Linderman RE, Cooper RF, Carroll J. Assessing Interocular Symmetry of the Foveal Cone Mosaic. Invest Ophthalmol Vis Sci (2020) 61(14):23. Epub 2020/12/18. doi: 10.1167/iovs.61.14.23.
9. Domdei N, Reiniger JL, Holz FG, Harmening W. The Relationship between Visual Sensitivity and Eccentricity, Cone Density and Outer Segment Length in the Human Foveola. Invest Ophthalmol Vis Sci (2021) 62:31. doi: 10.1167/iovs.62.9.31.
10. Reiniger JL, Domdei N, Holz FG, Harmening W. Human Gaze Is Systematically Offset from the Center of Cone Topography. Curr Biol (2021) 31:1-6. doi: 10.1016/j.cub.2021.07.005.
11. Wynne N, Cava JA, Gaffney M, Heitkotter H, Scheidt A, Reiniger JL, et al. Intergrader Agreement of Photoreceptor Topography at the Foveal Center on Adaptive Optics Scanning Light Ophthalmoscopy. Biomed Opt Express (2022) 13(8):4445-54. doi: 10.1364/BOE.460821.
12. Heitkotter H, Allphin MT, Untaroiu A, Min H, Warr E, Wynne N, et al. Peak Cone Density Predicted from Outer Segment Length Measured on Optical Coherence Tomography. Curr Eye Res (2023) 26:1-11.
13. Domdei N, Ameln J, Gutnikov A, Witten JL, Holz FG, Wahl S, Harmening WM. Cone Density Is Correlated to Outer Segment Length and Retinal Thickness in the Human Foveola. Invest Ophthalmol Vis Sci (2023) 64(15):11.

Supplementary Table 3. Intra-participant Differences in Density Estimates		
Tukey's Multiple Comparisons Test	Mean Difference (cones/mm²)	Adjusted P Value
5 cones vs. 10 cones	12,262	0.0038
5 cones vs. 15 cones	20,924	<0.0001
5 cones vs. 20 cones	25,599	<0.0001
5 cones vs. 40 cones	32,296	<0.0001
5 cones vs. 60 cones	34,852	<0.0001
5 cones vs. 80 cones	36,781	<0.0001
5 cones vs. 100 cones	38,538	<0.0001
5 cones vs. 150 cones	41,454	<0.0001
5 cones vs. 200 cones	43,773	<0.0001
10 cones vs. 15 cones	8,662	0.1459
10 cones vs. 20 cones	13,337	0.0009
10 cones vs. 40 cones	20,034	<0.0001
10 cones vs. 60 cones	22,590	<0.0001
10 cones vs. 80 cones	24,519	<0.0001
10 cones vs. 100 cones	26,276	<0.0001
10 cones vs. 150 cones	29,191	<0.0001
10 cones vs. 200 cones	31,511	<0.0001
15 cones vs. 20 cones	4,675	0.8913
15 cones vs. 40 cones	11,372	0.0108
15 cones vs. 60 cones	13,928	0.0004
15 cones vs. 80 cones	15,857	<0.0001
15 cones vs. 100 cones	17,614	<0.0001
15 cones vs. 150 cones	20,530	<0.0001
15 cones vs. 200 cones	22,849	<0.0001
20 cones vs. 40 cones	6,697	0.4925
20 cones vs. 60 cones	9,253	0.0900
20 cones vs. 80 cones	11,182	0.0134
20 cones vs. 100 cones	12,940	0.0016
20 cones vs. 150 cones	15,855	<0.0001
20 cones vs. 200 cones	18,174	<0.0001
40 cones vs. 60 cones	2,556	0.9982
40 cones vs. 80 cones	4,485	0.9138
40 cones vs. 100 cones	6,243	0.5960
40 cones vs. 150 cones	9,158	0.0976
40 cones vs. 200 cones	11,477	0.0096
60 cones vs. 80 cones	1,929	0.9998
60 cones vs. 100 cones	3,687	0.9745
60 cones vs. 150 cones	6,602	0.5141
60 cones vs. 200 cones	8,921	0.1188
80 cones vs. 100 cones	1,758	>0.9999
80 cones vs. 150 cones	4,673	0.8915
80 cones vs. 200 cones	6,992	0.4271
100 cones vs. 150 cones	2,915	0.9952
100 cones vs. 200 cones	5,234	0.8057
150 cones vs. 200 cones	2,319	0.9992

Supplementary Table 4. Results by Sampling Window Size

Window Size (# of cones)	PCD (cones/mm²)	PCD (cones/deg²)	Density at the CDC (cones/mm²)	Density at the CDC (cones/deg²)	Average PCD-CDC Location Offset (µm)	Average Window Size Bound Area (µm²)
5	229,500	19,489	180,157	15,273	19.27	45.98
10	212,648	18,060	175,567	14,870	17.79	92.58
15	205,684	17,467	177,265	15,014	15.85	138.93
20	201,646	17,121	177,901	15,060	17.09	185.50
40	193,399	16,414	176,352	14,940	15.98	370.96
60	189,881	16,118	175,390	14,857	15.71	555.97
80	187,558	15,921	174,996	14,828	16.35	740.71
100	185,501	15,747	174,696	14,804	16.91	925.05
150	182,184	15,465	174,295	14,776	16.06	1384.26
200	179,614	15,245	174,044	14,758	16.63	1841.14
